# Emerging roles of noncoding RNAs in human cancers

**DOI:** 10.1007/s12672-023-00728-w

**Published:** 2023-07-13

**Authors:** Shijie Wu, Yiwen Wu, Sijun Deng, Xiaoyong Lei, Xiaoyan Yang

**Affiliations:** 1 School of Pharmaceutical Science, Hengyang Medical College, University of South China, 28 Western Changsheng Road, Hengyang, 421001 Hunan People’s Republic of China; 2 Hunan Provincial Key Laboratory of Tumor Microenvironment Responsive Drug Research, University of South China, 28 Western Changsheng Road, Hengyang, 421001 Hunan People’s Republic of China

**Keywords:** ncRNA, miRNA, circRNA, lncRNA, Cancer

## Abstract

Studies have found that RNA encoding proteins only account for a small part of the total number, most RNA is non-coding RNA, and non-coding RNA may affect the occurrence and development of human cancers by affecting gene expression, therefore play an important role in human pathology. At present, ncRNAs studied include miRNA, circRNA, lncRNA, piRNA, and snoRNA, etc. After decades of research, the basic role of these ncRNAs in many cancers has been clear. As far as we know, the role of miRNAs in cancer is one of the hottest research directions, however, it is also found that the imbalance of ncRNAs will affect the occurrence of gastric cancer, breast cancer, lung cancer, meanwhile, it may also affect the prognosis of these cancers. Therefore, the study of ncRNAs in cancers may help to find new cancer diagnostic and treatment methods. Here, we reviewed the biosynthesis and characteristics of miRNA, cricRNA, and lncRNA etc., their roles in human cancers, as well as the mechanism through which these ncRNAs affect human cancers.

## **Introduction**

Proteins in the human body are obtained by transcription and translation of RNA, but studies have shown that only 2–3% of the genes in the human body can encode proteins [1, 2]. The rest are ncRNAs that do not code for proteins, which were once considered “useless” by scientists. Since lin-4 and let-7 were found in Cryptorhabditis elegans more than 20 years ago, the research on ncRNAs has started [3]. More than ten years ago, after the role of microRNA (miRNA) in cancer was clearly understood, the research on ncRNA ushered in a new round of upsurge. After years of research and exploration, many different ncRNAs have been found in a variety of cancers. Non-coding RNAs include miRNAs, cricRNAs, lncRNAs, snRNAs, and piRNAs and so on [4]. These ncRNAs vary in length. According to length, small ncRNAs are those with less than 200 nucleotides, while long ncRNAs have more than 200 nucleosides, including lincRNAs, NATs, and T-UCRs [4]. After a long time of research, scientists’ views on ncRNA have changed greatly. Studies have found that many types of ncRNAs play a dynamic role in the regulation of transcription and translation, and participate in the occurrence and development of many human cancers [4]. NcRNAs usually act on targets or signal pathways to inhibit or promote the development of cancers, and can also judge the type and development process of diseases by observing the type and stage of ncRNAs in cancers. The research on different kinds of ncRNA cannot be carried out alone. The action mode of ncRNAs is complex. In addition to acting on the target alone, it can also be a synergistic effect of multiple ncRNAs on a target. For example, circRNA can be used as a “miRNA sponge” to inhibit the binding of miRNA to the target and further have other effects [4]. This review summarized the biosynthesis and characterization of several ncRNAs, and their impact on several human cancers. In this review, we also discussed the challenges faced by ncRNA-based therapies and possible solutions.

## Biosynthesis and characteristics of ncRNA

### miRNA

MiRNA is one of the ncRNAs that are 22 nucleotides in length and can participate in many biological processes, such as cell cycle, differentiation, development, and metabolism via post-transcriptionally regulate gene expression, and its evolutionarily conserved single-stranded RNA [1, 4–6]. Abnormal regulation of miRNAs can lead to many human cancers, such as gastric cancer, breast cancer, lung cancer and so on [1]. 70% of miRNAs are located in exons or exons of protein-coding genes, the rest are located in intergenic regions [7]. The biosynthesis of miRNAs can be divided into the following steps: it was first transcribed by polymerase II into primary transcripts (pri-miRNA) of up to several thousand bases, and the pri-miRNA hold into hairpins, which act as substrates for two members of the RNase III family of enzymes, Drosha and Dicer. It is then processed by the enzyme Drosha in the nucleus into precursor strands (pre-miRNA) of about 70 nucleotides [8]. Subsequently, pre-miRNA is exported to the cytoplasm via export-5, where it is cleaved by RNase Dicer into a double-stranded miRNA, then the double-strand is separated by helicases and the mature strand is bound to RNA-induced silencing complex (RISC) as part of it [9]. After the duplex is formed, the one with unstable base pairing at the 5’ end is more likely to be selected for binding to RISC, while the other is degraded [10]. During miRNA maturation, many cofactors or accessory proteins have important regulatory effects on Drosha and Dicer. For example, arsenite-resistance protein 2 (ARS2) supports Drosha processing of pri-miR-21, pri-miR-155, or pri-let-7, providing functional coupling of pri-miRNA transcription and processing [11]. The p68 and p72 helicases are one of the Drosha Microprocessor complex components, which can stimulate the processing of one-third of murine pri-miRNAs [9]. Transporters mediate the transport of mature miRNAs between the cytoplasm and nucleus. Importin8 (IPO8), a member of the nuclear protein beta family, was found to play an important role in mediating the cytoplasmic-nuclear transport of mature miRNAs, which requires the AgO2 complex [12]. MiRNAs typically promote mRNA degradation or the inhibition of translation initiation through incomplete pairing with the 3’-UTR bases of target mRNAs, ultimately resulting in translational repression of the mRNA, resulting in reduced protein export [13]. MiRNAs not only play a role in cells but also in blood. In addition, a miRNA can regulate multiple targets, and multiple mRNA targets can also be regulated by a miRNA [1].

### cricRNA

CircRNAs are gene-regulatory RNA transcripts with covalently closed circular structures that are highly stable, especially in neural tissues circRNAs [14, 15]. This high stability may be due to their covalently closed ring structure which protects them from exonuclease-mediated degradation [16]. However, in highly proliferative tissues, cricRNA may be downregulated, possibly due to dilutional diffusion by proliferation before reaching a plateau [17, 18]. Most circRNAs (84%) are derived from protein-coding genes and are generated by a special form of alternative splicing called backsplicing-pre-mRNAs connect the 3’splice site of the downstream exon to the 5’splice site of the upstream exon backsplicing [19–21]. All exons can be backspliced except the first and last exons [22]. The formation of initiating cricRNA requires specific genomic features: First, the exons and flanking introns of cricRNA must be very long, usually 3 times longer than standard lincRNA. Then, these introns must contain reverse complementary sequence elements [23]. In addition to this mechanism, there are other mechanisms for the biogenesis of cricRNA, which still need to be further studied. The transport of circRNAs from the nucleus to the cytoplasm often takes the following ways. By the ATP-dependent RNA helicase DDX39A (also known as the nuclear RNA helicase URH49 or URH49) and the spliceosome RNA helicase DDX39B (also known as the dead box protein UAP56 or UAP56) in a size-dependent manner [24]. In human cells, UAP56 exports long cricRNA (> 1200 nucleotides), while URH49 exports short circRNAs (< 400 nucleotides) [24]. However, different species may have different length requirements for exporting circRNAs from the nucleus to the cytoplasm [25]. The degradation of cricRNA is less known at present, and some studies have shown that cricRNA can be degraded by RNase L [14, 26]. CircRNAs can exert biological functions by regulating protein function or self-translation, or acting as microRNA inhibitors “sponge” [24, 27–29].

### lncRNA

LncRNAs are ncRNA greater than 200 nucleotides, which is relatively abundant in human body. Now thought to play a key role in many cellular processes [30], including cell cycle, differentiation and metabolism [31, 32]. LncRNAs are not evolutionarily highly conserved. Often originating from intergenic regions, lncRNA transcripts are often “like-mRNAs”, because it is transcribed by RNA polymerase II and is capped and polyadenylated, contain typical splicing sites, and (Gu, Ag) introns/exons are also similar in length to mRNA [22, 30, 33–35]. Although the expression level of lncRNA is usually lower than that of mRNA, its specificity is strong [36, 37]. The lncRNA was originally thought to be unstable, but can be stabilized by polyadenylation [38] and non-polyadenylated can be stabilized by secondary structure [39]. The main functions of lncRNA are regulation of transcription, epigenetic modification, protein, RNA stability, translation and post-translational modification [30]. Recently shown to interact directly with signaling receptors [40]. It can function in three main ways: Firstly, interact with other components in cells such as DNA, RNA and proteins. Secondly, gene regulatory elements are embedded in lncRNA gene transcripts, and the activity of regulatory elements is determined by the activity of lncRNA genes. Thirdly, the transcription process affects the genome and thus gene activity [41].

### Other ncRNA

PiRNA is a single-stranded RNA that interacts with P-element-induced wimpy testis (PIWI) proteins and its about 26–31 nucleotides in length, the sequence is not conserved [42–45]. First discovered in germ cells, and the 3’ end shows a 2-o-methyl modification [46]. PIWI protein is a subtype of Argonaute protein. Compared to other known cellular RNAs, piRNAs display a different nucleotide sequence [47]. Its synthesis is as follows: First transcribed into long transcripts by RNA polymerase II, then exported to the cytoplasm and processed into smaller sequences (mature PIRNAs) by unknown protein complexes in a still unclear Dicer-independent manner [44]. Its functions include affecting transposon silencing, spermatogenesis, Gen Ome rearrangements, epigenetic regulation, protein regulation, and maintenance of germ cells [43]. PIWI protein is associated with cancers development [43, 46], two isoforms of PIWI proteins—PIWI1 and PIWI2, of which overexpression of PIWI1 is associated with cell cycle arrest and overexpression of PIWI2 is associated with anti-apoptotic signaling and cell proliferation [1].

SnRNA is transcribed by RNA polymerase II (pol II), highly expressed in human and drosophila cell cycle and cell development, and usually exists in clusters in the genome [48]. SnRNA can be divided into Sm and Lsm classes based on shared sequence characteristics and protein cofactors. Sm classes include U1, U2, U4, U4atac, U5, U7, U11, U12. Lsm classes include U6, U6ata [49]. SnRNA differs from protein coding genes in some aspects, such as the fact that the transcript of the snRNA gene is not spliced and the 3 ‘- terminal is not phosphorylated, which may be to prevent translation [50]. There are also some specificity, with snRNA gene specificity 3 ‘- box located 9-19 bp downstream of the RNA coding region [50]. It can also regulate some specific expressions, such as U1snRNA specific expression in the human brain [49]. However, there are also some similarities between snRNA and mRNA, and pol II dependent snRNA also requires universal factors TATA binding protein (TBP), transcription factors IIB (TFIIB), TFIIA, TFIIE, and TFIIF for in vitro transcription [50].

In the nucleus, some snRNAs are identified as promoters of mRNA splicing and have nucleolar specificity and are named small nucleolar RNA (snoRNA)[51].Approximately 60–300 nucleotides in length[52].The main functions of snoRNA include influencing the methylation and pseudouridine of rRNA, selective splicing of mRNA, and telomere synthesis [52]. Classic snoRNAs can be divided into three categories, the C/D box snoRNA(SNORD), the H/ACA box snoRNA, and the small cajal body specific RNAs(SCARNAs) [51]. SNORD and SNORA are usually located in the nucleolus, where they modify rRNA together with RNP, while SCARNA is located in the cajal body, which can promote U1 modification to U6 [51].At the beginning, researchers believed that the activity of snoRNA was limited to nucleolus, targeting the post transcriptional modification of ribosomal RNA (rRNA), thus supporting the production of ribosome ribonucleoprotein (RNA) complexes. However, the results showed that snoRNA not only affects nucleolus, but also affects nucleus and even cytoplasm, thus affecting some diseases[51]. SnoRNA can affect genetic disorders such asPrader-Willi syndrome (PWS) and Angelman syndrome (AS) [51].

tRNA-derived small RNA(tsRNA)mostly produces stress conditions such as viral infection, ultraviolet radiation, oxidative stress, etc[49]. tSRNA can be classified into three categories based on the cleavage positions of mature or precursor tRNA transcripts, tiRNA9also named tRNA halves),tRNAfragments (including 5′, 3′ and inter tRF (i-­tRF) and 3′U tRNA fragments (3′U tRFs)[49]. The length of tiRNA is 28–36 nucleotides, which is produced by cutting the anti codon ring of mature tRNA by ribonuclease [49]. The length of tRNA fragments is 15–32 nucleotides, which is located in the D of tRNA- β Ring or T- β Produced by cutting in the ring. And the length of 3 ‘U tRNA fragments is 15–32 nucleotides, which are produced during the maturation process of tRNA by RNaseZ enzyme cleavage of the 3’ end of tRNA precursor [49]. Research has shown that tsRNA can interact with Ago and Piwi proteins, which may affect gene expression regulation at both pre and post transcriptional levels[53].

## The role of ncRNAs in cancers

**Fig. 1:**
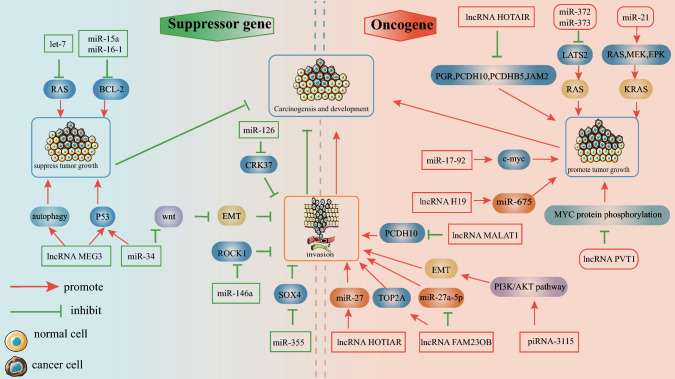
ncRNAs in cancers ncRNAs in cancer can affect cancer initiation, progression via three ways: act as oncogenes, cancer suppressors or affect cancer metastasis. When it is used as oncogene, lncRNA H19, miR-21 and miR-17-92 mainly promote the target, while lncRNA PVT1, miR-372/373 and lncRNA HOTAIR inhibit the target. As tumor suppressor, miR-34 and lncRNA MEG3 both promote target P53, and lncRNA MEG3, it can affect autophagy and inhibit cancer, and miR-15a and miR-16-1 affect BCL-2 and inhibit cancer. When lncRNA plays a role in influencing tumor metastasis, it affects the target, thus affecting the occurrence of cancer

Dysregulation of ncRNAs can lead to a variety of human cancers. At present, some mechanisms have been clear, and most of the mechanisms still need to be further studied (as shown in Fig. 1).

### ncRNA and cancer

In China, the incidence of cancer tends to be higher, and treatment is often ineffective due to late detection and higher rates of metastases [54]. The process of malignant transformation of normal cells into cancers is called tumorigenesis and involves the activation of proto-oncogenes into oncogenes. Proto-oncogenes refer to genes that can normally control cell division, apoptosis, and differentiation. Chromosomal translocation, gene mutation [55] and gene amplification can lead to proto-oncogene activation [56, 57]. For example, a study mapped the insulating neighbors of T-cell acute lymphoblastic leukemia (T-ALL) and found that cancer cell genomes contain recurring microdeletions that eliminate insulating neighbors containing prominent T-ALL proto-oncogenes. Area border site, this leads to the conclusion that proto-oncogenes can be activated by disrupting genetic alterations in insulating neighborhoods in malignant cells [58]and inactivation of tumor suppressor genes, which are genes that inhibit malignant lesions of normal cells [57, 59]. Loss of tumor suppressor gene function is caused by deletion or inactivation of both alleles [60]. The occurrence of cancer is a gradual process. During this process, the proliferation of cancer cells is out of control, the apoptosis mechanism is changed, and normal cells are infiltrated, causing cancer metastasis. Cancer metastasis is one of the main causes of death. After cancer metastasis, the risk factor and the difficulty of treatment will increase [61].

NcRNAs are often considered to be oncogenic factors and tumor suppressors in various major cancers[22], the role of ncRNAs in cancers is often not single, and the interaction of multiple different ncRNAs can also regulate some important cellular programs, including in cancers [3]. Traditional therapeutic drugs include cytotoxic drugs, antimetabolites and, some hormonal drugs. The toxicity of treatment methods using these drugs poses a huge challenge to tolerance and compliance and is often accompanied by various side effects, some of which are unbearable for patients, such as gastrointestinal reactions, nephrotoxicity, bone marrow suppression, etc. [62–64]. Research on ncRNA can also develop new cancer treatment methods, which are expected to improve patient compliance, improve efficacy and reduce side effects.

#### miRNA and cancer

MiRNAs are often located in fragile regions of chromosomes [1]. Abnormally expressed in almost all human cancers, abnormally expressed miRNAs can massively disrupt cell signaling pathways, thus having a profound impact on the occurrence and development of cancers [7]. Specific regulation can be manifested in the regulation of key gene expression in the cancer cell cycle, apoptosis, and migration [65–68]. Loss of miRNA function can be caused by a variety of mechanisms, including genetic mutations, deletions, epigenetic silencing, and alterations in miRNA processing [69]. Aberrant miRNAs have diagnostic, prognostic, and therapeutic implications-miRNAs are differentially expressed in different cancers, thereby distinguishing different cancers [47]. The effect on cancers is mainly manifested in the following three ways: Firstly, miRNA as a tumor suppressor. Secondly,, miRNA as an oncogene. Thirdly, miRNA promotes or inhibits cancer metastasis [70, 71]. However, the specific functions of miRNAs should be comprehensively analyzed according to the pathological type and physiological environment in which they are located, sometimes, they are “tumor inhibitors”, and sometimes they are “oncogenes”. Like miR-29, it helps stop disease progression in chronic B-chronic lymphocytic leukemia(B-CLL), acting as a tumor suppressor at this time but is also elevated in acute myeloid leukemia and more aggressive B-CLL, implying that this miRNA can also function as oncogenes [22, 72].

The existence of some miRNAs can inhibit the malignant transformation of normal cells, which can be called tumor suppressors. Compared with normal cells, their expression is often down-regulated in cancers [7, 73, 74] (as shown in Table 1). Let-7 is mostly located in vulnerable gene regions related to lung cancer, breast cancer, and cervical cancer, and is often down-regulated in these cancers. Let-7 can inhibit the RAS family, one-third of human cancers are associated with this oncogene [75]. MiR-15a/miR-16-1 is located in the cytoplasmic 13q14 region and is downregulated in chronic lymphocytic leukemia(CLL) patients [7]. MiR-34 is the first tumor suppressor shown to synergize with the tumor suppressor gene p53 to regulate cancers [76]. There are mainly three family members, miR-34a, miR-34b, and miR-34c. Among them, miR-34a is located on chromosome 1p36.22 and has a unique transcript, while miR-34b/c is located on chromosome 11q23.1 and shares a transcript. The expression level of miR-34a in rectal cancer is lower than that of surrounding non-cancer cells, and miR-34a and miR-34b can also inhibit cancer cell metastasis and invasion [77], miR-34 is downregulated in the prostate and can negatively regulate the Wnt signaling pathway, inhibiting EMT (a cellular biological process in which epithelial cells lose their morphological and adherent ability to acquire a mesenchymal phenotype) associated with migration and invasion. In addition, miR-34 is downregulated in breast cancer, osteosarcoma, and multiple myeloma [72, 76, 77].

**Table 1:** MicroRNA in cancer

MicroRNA	Location	Targets	Expression	Roles	References
Let-7	Vulnerable intergenic regions associated with cancer.	RAS, HMGA2	Downregulated in lung and breast cancer.	TS	[7, 65]
miR-15a miR-16-1	Chromosome13q14 Region	BCL-2	Upregulated in CLL	TS	[7]
miR-29	Chromosome 7q23, Chromosome1q32	MCL-1	Downregulated in CLL, lung cancer. etc.	TS	[7]
miR-34a/b/c	miR-34a is located in Chromosome 1p36.22, miR-34b/c is located in chromosome 11q23.1	P53 Wnt	Downregulated in rectal, breast and prostate cancer, and inhibits tumor invasion and migration.	TS	[2, 62, 73]
miR-155	Chromosome 21q23	c-maf	Upregulated in lung and breast cancer, CLL, AML.	OG	[22]
miR-21	Intimal region of the TMEM49 gene	Ras MEK EPK	Upregulated in pancreatic and liver cancer, glioblastoma.	TS	[70, 71]
miR-17-92 cluster	Chromosome 13q31.3	C-myc	Upregulated in Solid tumors and hematological malignancies.	OG	[73]

*TS* tumor suppresor, *OG* oncogenes

When miRNAs act as oncogenes, they can promote abnormal cell growth, promote cancer formation, or directly inhibit the activity of tumor suppressors. Generally upregulated in cancer cells [73, 78] (as shown in Table 1). For example, miR-155 can promote the proliferation of abnormal B cells, leading to lymphoma [22, 79]. MiR-21 is one of the most well-characterized oncogenic miRNAs and is up-regulated in almost all types of cancers [4]. Multiple experiments have demonstrated the oncogenic function of miR-21. A study indicates miR-21 overexpression induces pre-B-cell lymphoma in mice [80, 81], promoting KRAS-dependent carcinogenesis by activating the Ras/MEK/ERK pathway [82]. MiR-17-92cistron is located in 13q31, up-regulated in follicular lymphoma, mantle cell lymphoma, primary cutaneous B-cell lymphoma, and other cancers. The miR-17-92 cluster cooperates with the oncogenic factor c-myc to jointly promote cancer development [83]. MiR-372/miR-373 can directly inhibit the tumor suppressor gene LATS2 to balance wild-type TP53, thereby interacting with RAS, synergistically inducing human primary cell proliferation and tumorigenesis—a mechanism involved in human testicular germ cell tumorigenesis, allowing oncogene growth by targeting the wild-type TP53 pathway [7, 84]. RAS is the second largest gene mutation driver in human cancers, and mutations in the RAS gene or its regulators allow the RAS protein to remain active [85]. RAS mutant proteins regulate cancer cells proliferation, apoptosis, metabolism, and angiogenesis through downstream MAPK, PI3K, and other signaling pathways [86].

In addition to directly promoting or inhibiting the occurrence of cancers, miRNAs can also affect the metastasis of cancers, thereby affecting the occurrence and development of cancers. Cancer metastasis involves multiple steps. Firstly, growth and enlargement of the primary tumor. Secondly, invades surrounding tissues and penetrates lymphatics and blood vessels. Thirdly, lymphatic and intravascular tumor thrombus forms and travels with lymph, and blood. Fourthly, stops in the walls of lymphatics or blood vessels in distant organs. Fifthly, breaks out of the lymphatic and blood vessel walls where it stops and invades surrounding tissue. Sixth, cancer cells proliferate, grow, and successfully metastasize here. In some studies, multiple miRNAs have been shown to promote cancer metastasis. The following is a partial summary. Upregulation of miR-10b, although not affecting cancer cell proliferation, promotes migration and invasion of breast cancer cells [87]. An experiment employed miRNA microarray analysis and LASSO logistic statistical model to identify major functional exosomal miRNAs. Invasion and scratch assays were performed to examine the migration and invasion of liver cancer cells. Experimental results show that exosomal miR-21 and miR-10b induced by the acidic microenvironment of liver cancer can promote cancer cell proliferation and metastasis [88]. In addition, miRNAs can also inhibit cancer metastasis, and miR-335 can inhibit breast cancer metastasis by directly inhibiting SOX4 expression [89]. SOX4 is a transcription factor that plays a role in the development and migration of cellular progenitors. MiR-126 can inhibit cell adhesion, migration, and invasion by inhibiting CRK37, a protein involved in actin remodeling, and adapter signaling proteins for focal adhesion formation and cell migration [89]. MiR-146a can directly reduce the expression of Rho-activated protein kinase Rock1. In addition to its important role in morphogenesis, Rock1 is involved in hyaluronan-mediated transformation and metastasis of hormone-refractory prostate cancer in vivo [90]. Therefore, miRNAs can be used as markers to distinguish metastatic cancer from non-metastatic cancer. For example, experiments show that miR-10b can distinguish metastatic melanoma from non-metastatic melanoma [91].

#### cricRNA and cancer

The circular structure of circRNA makes it a stable structure, so it can exist stably in peripheral body fluids such as plasma and saliva [16]. CricRNA can bind to corresponding miRNAs or directly bind to proteins to regulate cancer energy metabolism [29]. One of the hallmarks of cancers is the change in energy metabolism, which can provide necessary nutrients for the occurrence and development of cancers [92, 93]. Alternatively, circRNAs can promote the metastasis and drug resistance of malignant cancers and serve as markers for cancer diagnosis and treatment. The enzymes required for sugar metabolism are as follows, the three rate-limiting enzymes include hexokinase (HK), 6-phosphate fructose-1-kinase (PFK), pyruvate kinase (PK), lactate dehydrogenase A (LDHA), and pyruvate dehydrogenase (PDH) of acetyl-CoA, etc. [93–95]. CricRNA can affect sugar metabolism by affecting various enzymes in sugar metabolism [93, 94]. For example, circRNA Circ-Amotl1 can physically bind to PDK1 and AKT1 and transfer to the nucleus to antagonize apoptosis [95]. Cichipk3 can sponge miR-124, and miR-124 represses the expression of several enzymes and transporters of glycolysis [28]. In addition to affecting glucose metabolizing enzymes, cricRNA can also affect glucose metabolism through other pathways, such as by affecting transcription factor metabolism and affecting glucose metabolism through signaling pathways [29]. In addition, cricRNAs can also affect lipid metabolism, alterations in lipid metabolism can affect cancer development, and increased lipolysis leads to a wasting syndrome known as cancer cachexia, characterized by the acute fat loss [96]. For example, cric-0046367 can bind miR-34a and protect peroxisome proliferator-activated receptor (PPAR)α from transcriptional repression. PPARα activates CPT2 and ACBD3 to degrade lipids [97]. In addition to affecting metabolism, cricRNA can regulate transcription and mRNA splicing through the interaction of RNA polymerase II and snRNA, and regulate protein localization and activity. The FBXW7-185AA protein encoded by glioblastoma cricRNA FBXW7 plays an important role in the occurrence and prognosis of glioblastoma, and its expression is down-regulated in glial tissue, and the survival of patients with high expression of cricRNA FBXW7 is lower than that of low expression of patients [98]. Circ-SHPRH encodes a functional protein SHPRH-146aa as a tumor suppressor in glioblastoma, and its expression is decreased in glioblastoma [99]. Forethmore, circURI1 is also as tumor suppressor, while cricRNA_0000285, circRNA WHSC1 and hsa_circ_001783 as oncogenes [100–105]. The effect of cricRNA on drug resistance is as follows, circAKT3(hsa_circ_0000199)derived from exons 8, 9, 10, and 11 of the AKT3 gene, which is highly expressed in cisplatin-resistant gastric cancer cells, and tissues and can promote cisplatin resistance in gastric cancer [106]. A novel circRNA, circFN1, also promotes cisplatin resistance in gastric cancer [107]. Paclitaxel (PTX) is an effective first-line chemotherapeutic agent in GC, but drug resistance diminishes its efficacy. CircRNA Circ-PVT1 promotes resistance of gastric cancer cells to PTX through miR-124-3P-mediated upregulation of ZEB1 [108]. The roles of other cricRNAs in cancers are shown in Table 2.

**Table 2:** Other cricrRNA and cancer

CricRNA	Cancer types	Mechanisms	Roles	References
cricCDDC66	Colon cancer	miRNA sponge	OG	[22]
cricHIPK3	Bladder cancer	miRNA sponge	TS	[22]
cricPCNXL2	Kidney cancer	miR-153sponge	OG	[23]
hsa_circ_001895	Kidney cancer	miR-296-5p sponge	OG	[23]
cric-AKT3	Kidney cancer	miR-296-3p sponge	TS	[23]
cricANRIL	Breast, bladder and gastric cancer	Impairs ribosome biogenesis leading to p53 activation	OG	[33, 99]
cricRNA_0000285	Cervical cancer	Upregulation of FUS increases CC proliferation and metastasis	OG	[90]
circRNA WHSC1	Endometrial and ovarian cancer	miR-646 sponge,miR-145 sponge, miR-1182 sponge	OG	[91, 92]
circURI1	Gastric cancer	Directly interacts with heterogeneous ribonucleoproteins to inhibit GC transfer	TS	[93, 94]
hsa_circ_001783	breast cancer	miR-20c-3p sponge	OG	[95]

*FUS* A ubiquitously expressed protein belonging to a family of heterogeneous nuclear proteins that plays important roles in DNA damage, cellular stress responses, RNA metabolism and processing. MiRNA sponge: It is a competitive inhibitor of miRNA, which can adsorb the corresponding miRNA and compete with miRNA target genes

#### lncRNA and cancer

Many lncRNAs exhibit cell or tissue tumor-specific expression, making them potential therapeutic targets. LncRNAs regulate gene expression in the nucleus by regulating epigenetic and transcriptional levels, and in the cytoplasm by regulating post-transcriptional and Translational regulation of gene expression [109]. LncRNAs can act as both tumor suppressors and oncogenes, just like miRNAs in cancers [110]. As an oncogene, HOTAIR is transcribed from the HOXC locus during normal development, and its overexpression can promote the development of gastric cancer [34]. Overexpression of HOTAIR can inhibit tumor suppressors such as progesterone receptor (PGR), protocadherin 10( PCDH10 ), protocadherin β5 (PCDHB5) and junctional adhesion molecule 2 (JAM2) thus showing a tumor-promoting effect [110, 111]. H19 is the first lncRNA found to be overexpressed in hepatocellular carcinoma and rectal cancer. It can affect the development of cancer through the following pathways. Since H19 is the precursor of miR-675, the increase of H19 leads to the increase of miR-675 and rectal cancer. The tumor suppressor retinoblastoma protein is reduced in cancers, which in turn promotes tumor proliferation. Or lncRNA acts as the ceRNA of several different miRNAs. The lncRNA acts as the “sponge” of different miRNAs, such as let-7, etc. [33]. GAS6-AS2 and FOXD-AS1 also act as oncogenes [112, 113]. In addition, lncRNAs can also regulate oncogenes, such as PVT1, an intergenic lncRNA derived from a polysplicing isoform of 8q24.21, which can inhibit MYC protein phosphorylation and increase its stability, thereby increasing tumorigenicity [114, 115]. Lung cancer-associated transcript 1 (LUCAT1), located in the antisense strand of the q14.3 region of chromosome 5, has been confirmed to be highly expressed in various malignant tumors through years of research and is involved in breast cancer, ovarian cancer, thyroid cancer, and other cancers [109]. LncRNAs can also act as tumor suppressors, such as ERRA (Telomeric Repeat-containing RNAs) is a group of lncRNAs transcribed from telomeres, about 100 bp-9 kb in size, which can act as a tumor suppressor and negatively regulate telomerase [116, 117]. MEG3 (Maternally Expressed 3) is a tumor suppressor located on chromosome 14q32.2 that is normally downregulated in cancer cells [118, 119]. Overexpression of MEG3 in bladder cancer cells induces autophagy, and in addition, MEG3 is involved in the accumulation of the tumor suppressor P53 [120, 121].NAMA and PTCSC3 also as tumor suppressors [122]. In addition to affecting the occurrence and development of cancers, the abnormal expression of lncRNA can also affect the treatment of cancers by drugs. For example, lncRNAs can affect the drug resistance of gastric cancer drugs. The lncRNA PCAT-1 is highly expressed in cisplatin-resistant gastric cancer tissues and cells. PCAT-1 epigenetically silences PTEN by binding to the histone methyltransferase enhancer of zeste homolog 2 (EZH2). Silencing counteracted PCAT-1 knockdown-mediated enhancement of cisplatin sensitivity in CDDP-resistant GC cells [123]. LncRNA HOTAIR can inhibit the expression of miR-217 and promote the resistance of gastric cancer cells to doxorubicin and paclitaxel, and its abnormal expression increases the proliferation, cell cycle, and migration of GC [124]. LncRNA ZFas1 enhances the resistance of gastric cancer cells to paclitaxel (PTX) by altering cell cycle-related proteins (cyclin D1, cyclin E, and cyclin B1) and Wnt/β-catenin signaling [125].

In addition to the above effects, lncRNA can also affect cancer proliferation, migration, etc. LncRNA MALAT1 can promote gastric cancer metastasis by inhibiting PCDH10 [126]. The lncRNA ARHGAP27P1 inhibits gastric cancer cell proliferation and cell cycle progression through epigenetic regulation of p15 and p16 [127]. LncRNA FAM230B promotes gastric cancer growth and metastasis by regulating the miR-27a-5p / TOP2A axis [128]. For more details, were listed in Table 3.

**Table 3:** Other lncRNA and cancer

LncRNA	Cancer types	Mechanism	Roles	References
GAS5	Breast cancer, glioblastoma, SCC	Affects GR signaling ,interacts with miRNA, reduces cell proliferation and increase apoptosis	TS	[22, 33]
LUNAR1	T-ALL	Activation of IGF1R expression in cis cells to regulate T cell growth	OG	[33]
NEAT1	Liver and ovarian cancer, melanoma	It is a target gene of P53 and can activate oncogenes	OG	[33]
LUCAT1	Kidney cancer	Promoting proliferation and invasion of clear cell renal cell carcinoma via AKT/GSK-3β signaling pathway	OG	[100]
GAS6-AS2	Bladder cancer	Induction of G1 arrest promotes tumor growth, and miR-298 sponge regulates CDK9 expression.	OG	[103]
FOXD2-AS1	Gastric cancer	Promote cell cycle progression and accelerate cell proliferation	OG	[104]
lncRNA-p21	Rectal cancer	Involved in the transcriptional regulation of P21 and P53	TS	[33]
NAMA	PTC	Regulated by the MAP pathway	TS	[113]
PTCSC3	PTC	Inhibit the expression of S100A4, VEGF and MMP9,inhibit the growth and invasion of TC cells	TS	[113]

*T-ALL* T cell acute lymphoblastic leukemia, *SCC* squamous cell carcinoma, *PTC* papillary thyroid cancer, *TC* thyroid cancer

#### Other ncRNA and cancer

In addition to popular ncRNAs such as miRNA and lncRNA, some uncommon ncRNAs can also affect the occurrence and development of cancer. Some studies have shown that this ncRNA is significantly different in cancer tissue and normal tissue, such as PIR-34,736, PIR-35407PIR-36,318, PIR-34377and PIR-31,106 is different between breast cancer and breast normal tissue [129]. PIR-651 is overexpressed in gastric, colon, lung, and breast tissues, and liver, mesothelioma, cervical, breast and lung cancer cell lines [130]. PiRNA-31,115 promotes cell proliferation and invasion in clear cell renal carcinoma through the PI3K/AKT pathway [131]. SnRNA can also regulate the occurrence of cancer. For example, U6snRNA is a key component of spliceosome RNA and the primary target of miR-10b in glioblastoma, which can regulate its development[132]. SnoRNA can compete with U11snRNA RNP, thereby altering the splicing of mRNA encoding E2F transcription factor (E2F7), which can lead to head and neck cancer and retinal cancer [51]. C/D boxsnoRNA U50 is down regulated in prostate cancer. When U50 expression rises, it can inhibit the formation of prostate cancer cell colonies, and other mutations and disorders of U50 may also affect the occurrence of breast cancer[133]. SNORA 42 is an H/ACA boxsnoRNA encoded in 1q22 that can affect the formation of nonsmall-cell lung cancer (NSCLC). Downregulation of SNORA 42 can induce cell apoptosis in vitro and reduce colony forming ability, as well as inhibit tumor formation in a mouse model [133]. Research has found that tsRNA-46 and tsRNA-47 are downregulated in CLL and lung cancer, indicating that tsRNA can act like piRNA by interacting with Piwi proteins. Therefore, tsRNA can interfere with the epigenetic regulation of genes [53].

## ncRNAs and other human diseases

MiRNAs are involved in the formation of neural development, dendrites and spines, and mutations in the central miRNA processing mechanism can cause various neurological diseases[1]. Seizures lead to differential miRNA expression, and human temporal lobe epilepsy and experimental epilepsy lead to changes in brain tissue-specific miRNA levels in a regional or even a neural compartment-dependent manner. For example, during epilepsy control, the expression of miR-132 changes[134]. LncRNA also plays an important role in neurodevelopment and the improvement of brain function. In the brain, about 40% are specifically expressed [135]. For example LncRNA AK037594 is only expressed in the dentate gyrus and hippocampus of the hippocampus CA1-3 region. MIAT (GOMAFU) is a nuclear-localized lncRNA that is only expressed in differentiated neural progenitor cells and a subset of postmitotic neurons [136]. The lncRNAs related to neural development are related to suz12, EST, and SOX2, indicating that lncRNAs are related to these proteins. If these lncRNAs are knocked out, neural differentiation will be impaired, indicating that lncRNAs play an important role in the regulation of neural development [135]. Huntington (HD) disease is caused by the elevation of 3 novel lncRNAs, and the lncRNA MIAT is down-regulated in schizophrenia patients. Fragile X tremor ataxia syndrome (FXTAS) and fragile X syndrome (FXS) are intellectual disabilities caused by an expansion of a CGG repeat in the 50-UTR of the FMR1 (a transcript of lncRNA) protein-coding gene [135].

NcRNA can participate in the innate antiviral immune response of host cells. In RNAi, miRNA can bind to the complementary sequence of the viral RNA strand to form a miRNA-induced silencing complex, which destroys the transcription of viral RNA and inhibits the expression of viral proteins [137]. Isoglycerides ameliorates depression by inhibiting NLRP3-mediated pyrophosphorylation via the miRNA-27a/SYK/NF-κB axis and also reduce UVB-irradiation-induced cell loss [138, 139].

The expression of ncRNA in glomerular cells is different from that in normal cells. In systemic lupus erythematosus, cri_0000479 is the most up-regulated cricRNA, which can be used to distinguish healthy people from patients with systemic lupus erythematosus and rheumatoid arthritis [23].

The pathogenesis of myotonic dystrophy is a CUG repeat within the 3’-UTR of the tensor kinase gene that binds to myotonic-like protein 1(MBNL1) and interferes with alternative splicing. The treatment for this disease is to target repetitive RNA and release MBNL1 from repeats using CUGrepeat-targeting [1].

In addition, abnormal expression of miRNAs in some monogenic diseases, such as miR-145 and miR-146 deletions, can lead to 5q syndrome. Mutations in miR-96 can cause deafness and also coordinate glucose and fat utilization in skeletal muscle and serve as a diagnostic marker for gestational diabetes [140, 141]. SnoRNA plays an important role in imprinting disorders, such as PWS syndrome, Angelman syndrome, etc. [1].

## ncRNA-based therapeutics

With the deepening of ncRNA research, many diseases have ncRNA-based therapies. Compared with traditional therapy, this kind of therapy has many advantages, such as the drug can be delivered to the target cells, and the effect of the drug is more accurate. Traditional antitumor drugs have more adverse reactions such as nausea and bone marrow toxicity, while ncRNA-based drugs have fewer adverse reactions and are more easily accepted by patients. A variety of approaches are currently available, and a few are briefly described below: oligonucleotide-based therapeutics have attracted much attention as potential therapeutics. Compared with small molecule drugs, it is easier to find molecules that inhibit the function of proteins [6]. Antisense oligonucleotide (ASOs) can act in two ways: Firstly, includes a DNA gap region flanked by chemically modified nucleotides to facilitate binding of complementary targets to increase nuclease resistance. Such interstitial ASOs form DNA-RNA hybrids with target mRNAs, recruit reverse transcriptase, and promote mRNA degradation. Secondly, absence of gaps, works by binding to target RNA sequences and blocking key proteins [6, 142, 143]. Specific methods include Modified mRNA (modRNA), siRNA or RNA inhibitors, small RNA mimics, etc [4, 144]. However, one of the disadvantages of this approach is off-target effects. Off-target effects are to the extent that they are not bound to the target, or bind to other irrelevant targets, which can be reduced by combined application, but still lack specific transmission [72]. There are also nuclease degradation, renal clearance, non-capillary skin adherence, and genotoxicity and delivery issues [145]. Chemical modification of nucleotides can increase stability and avoid enzymatic degradation, For example, the 2’-O-methyl modification of nucleotides can increase the resistance of oligonucleotides to nucleases; or the use of sulfur atoms to replace the non-bridging oxygen in the phosphate backbone to form phosphorothioate bonds can also reduced cleavage by nucleases, but membrane permeability decreases after chemical modification, limiting its application in vivo [145]. The use of various carriers not only solves the above problems, but also does not interfere with membrane permeability [145]. Such as the use of viral vectors, which are the most common, including lentiviral retroviruses and adenoviruses, the advantages of using this vector are stability, durability, and safety [146]. For example, using recombinant adeno-associated viral vectors with high affinity for the myocardium to directly target the heart [147]. Or use lipid carriers, which are used non-nucleic acid transfection viral vectors with high efficiency, but cationic lipids have poor stability and high toxicity in serum [148]. And polymer carrier, and then a nanocarrier with low immunogenicity, low cytotoxicity, distinguishable components and stable structure [72]. Among them, heart-targeting drugs can directly target the heart using a recombinant adeno-associated virus. Inhibition of miR-25, which is upregulated in heart failure, by nanoparticle-coupled antagonists of the delivery system [147]. LncRNA-based methods include post-transcriptional knockout of pathogenic RNAs through the RNA pathway, structurally inhibiting lncRNA functions (lncRNAs can form secondary or tertiary structures, and folds can also become targets), through spatial closure of promoters or Regulation of lncRNAs using genome editing technology [33, 149].

## Conclusion and prospect

This article mainly introduces the influence of ncRNAs on the occurrence and development of several human cancers. NcRNA plays a very important role in cancers, and people are more and more interested in ncRNAs affected by human cancers. Therapists based on ncRNAs also encourage scientists to constantly explore the mechanism of ncRNAs playing their pathological role, but this is a long way to go, and the known mechanism is no more than the tip of the iceberg, which still needs continuous efforts. As the saying goes, “The road is long, and I will go up and down.“ One of the main challenges for further research is to identify functional ncRNAs in the human genome. The current second-generation sequencing methods, such as RNA sequencing, will provide detailed information on the ncRNA transcriptome [1]. In addition, ncRNAs can be folded into a complex secondary structure, which further challenges clarifying the functions of ncRNAs. A clear understanding of the structure of ncRNAs is essential to identify the role of ncRNAs in disease. As mentioned above, the same miRNA can not only be used as a tumor inhibitor but also as a carcinogen. Humans have realized that there is an imbalance of ncRNAs in the disease and that some ncRNAs can be used to determine the prognosis, such as the risk of metastasis and the response to chemotherapy, so they are eager to develop a new treatment method. However, the development of new methods is not achieved overnight. There is still a long way to go to overcome many challenges. This new treatment is expected to overcome the shortcomings of traditional drugs, such as drug resistance. As mentioned earlier, the existence of multiple ncRNAs can affect the drug resistance of anti-cancer drugs. It is still worth exploring whether drug resistance can be reduced against these ncRNAs. At present, the drugs based on ncRNAs are mainly cancer drugs. In addition, drugs for cardiovascular diseases, nervous system diseases, etc. are under development and have achieved little. It is believed that phased results will be achieved in the near future.

## Data Availability

Not applicable.

## Abbreviations

RISC: RNA-induced silencing complex
ARS2: Arsenite-resistance protein 2
IPO8: Importin8
PIWI: P-element-induced wimpy testis
pol II: Polymerase II
TBP: TATA binding protein
TFIIB: Transcription factors IIB
snoRNA: Small nucleolar RNA
SNORD: The C/D box snoRNA
SNORA: The H/ACA box snoRNA
SCARNAs: The small cajal body specific RNAs
rRNA: Ribosomal RNA
PWS: Prader-Willi syndrome
AS: Angelman syndrome
T-ALL: T-cell acute lymphoblastic leukemia
B-CLL: T-cell acute lymphoblastic leukemia
CLL: Chronic lymphocytic leukemia
HK: Hexokinase
PFK: 6-phosphate fructose-1-kinase
PK: Pyruvate kinase
LDHA: Lactate dehydrogenase A
PDH: Pyruvate dehydrogenase
PPAR: Peroxisome proliferator-activated receptor
PTX: Paclitaxel
PGR: progesterone receptor
PCDH10: Protocadherin 10
PCDHB5: protocadherin β5
JAM2: Junctional adhesion molecule 2
LUCAT1: Lung cancer-associated transcript 1
MEG3: Maternally expressed 3
EZH2: Enhancer of zeste homolog 2
PIWI: P-element-induced wimpy testis
HD: Huntington
FXTAS: Fragile X tremor ataxia syndrome
FXS: Fragile X syndrome
E2F7: E2F transcription factor
NSCLC: Nonsmall-cell lung cancer
MBNL1: Myotonic-like protein 1
ASOs: Antisense oligonucleotide
